# Quality of Life in Iranian Patients with Acne

**DOI:** 10.1155/2012/571516

**Published:** 2012-02-15

**Authors:** H. Safizadeh, S. Shamsi-Meymandy, A. Naeimi

**Affiliations:** ^1^Neurosciences Research Center, Kerman University of Medical Sciences, Kerman 7616914115, Iran; ^2^Department of Dermatology, Afzalipour Medicine School, Kerman University of Medical Sciences, Kerman 7616914115, Iran; ^3^Medical Student Research Committee, Kerman University of Medical Sciences, Kerman 7616914115, Iran

## Abstract

Acne is a chronic inflammatory disease of pilosebaceous units. Although the acne isnot a life threatening disease, studies have revealed that it has significant effect on self-image and quality of life. The purpose of this paper was to investigate the health-related quality of life in patients with acne in an Iranian context. Dermatology Life Quality Index (DLQI) and Cardiff Acne Disability Index (CADI) were used for measuring quality of life, and severity of acne was measured by Global Acne Grading System (GAGS). The mean (±SD) of DLQI and CADI scores was 6.42 (±4.77) and 5.97 (±2.97), respectively. Acne influenced the quality of life in 51.8% of patients from moderate to very much, and the quality of life was affected by the severity of acne (*P* < 0.01). Since acne has significant effects on patient's quality of life, the management of patients with acne requires more attention to different aspects of quality of life.

## 1. Introduction

Acne vulgaris is the most common chronic inflammatory pilosebaceous disease that presents with formation of comedone, papule, pustule, and nodule. Lesions appear mostly on the face, but neck, chest, upper parts of back, and shoulders may also be involved. It is the most common dermatologic disease in adolescence and adulthood with similar incidence in both genders. The age peak of acne is 17 years old, and in 3% of males and 12% of females, it continues beyond twenty five, while some patients (1% of males and 5% of females) carry it into their forties [1].

This disease is often misunderstood as a simple puberty-related condition by common people and even medical community, while scientific evidence revealed that acne affects patients more than a simple dermatologic disease [2]. It has been observed that social and psychological impacts of acne are sometimes so complicated that cause serious problems in patients' body image, self-esteem, and socialization and even may lead to feel of anger [3]. Although it is not a life threatening condition, studies have shown that it has serious effects on body image of affected individuals leading to anxiety, depression, and social dysfunction [4, 5]. It has been well recognized that in regard to social and psychological outcomes, acne is comparable with disabling diseases such as asthma, epilepsy, diabetes, back pain, or arthritis [6].

Dermatologic diseases affect patients' life by causing pain, itching, disability in daily activities, psychic pressures (low self-esteem, nervousness), problems in social relationships, family problems, and treatment-related problems such as drugs side effects and imposed treatment costs and time [7]. This is true for acne as well and affects patients' quality of life. It specially has negative impact on emotions, interpersonal relationships, physical activities, social life, and professional statues [8]. Therefore, attention to the quality of life of these patients is of a great importance. As in different cultures the impact of disease on quality of life differs [9], the aim of present study was to investigate the quality of life of patients with acne in an Iranian context.

## 2. Materials and Methods

In this cross-sectional study conducted in Kerman—the largest province of Iran—220 consecutive patients with acne vulgaris referred to the dermatology clinics of Kerman city were investigated. Patients with other types of acne were excluded from the study. This work was approved by Ethics Committee.

Global Acne Grading System (GAGS) was used to grade disease severity in patients [10]. This grading system has six locations including five locations on the face (forehead, right cheek, left cheek, nose, and chin) and one on chest/back. For each location based on the surface area and distribution and density of pilosebaceous units, a factor was considered. Each of these six locations was graded separately from 0 to 40, and for measuring severity in each location, the factor was multiplied in the grade of that location and the final score was calculated by adding scores. Score of 1–18 was considered as mild acne, 19–30 as moderate, 31–38 as severe, and above 39 as very severe [10].

Dermatology Life Quality Index (DLQI) [11] and Cardiff Acne Disability Index (CADI) [12] were used to determine the quality of life of acne patients. DLQI is a general questionnaire for evaluation of quality of life in dermatology patients and is consisted of 10 questions about disease symptoms, feelings, daily activities, type of clothing, social or physical activities, exercise, job or education, interpersonal relationships, marriage relationships, and treatment. Its domain is from zero (without any effect on quality of life) to 30 (high effect on quality of life), and higher score shows worse quality of life. According to the score obtained through this instrument, the effect of disease on quality of life can be divided into 5 classes of without effect, low effect, moderate effect, high effect, and severe effect [11].

CADI questionnaire is specific for acne and contains 5 questions—relating to the last month—about feelings, interference with social life and interaction with the opposite gender, avoidance of public facilities, appearance of the skin, and perceived severity of disease status. Each question has 4 choices and is scored from 0 to 3 leading to the total score of 0–15. Higher score shows worse quality of life [12]. The questionnaires were completed anonymously, after assuring the responders about the confidentially of the data and explaining the objectives of the research.

Both questionnaires have Persian equivalents with confirmed reliability and validity [13, 14]. Data gathered by questionnaires were analyzed using *t*-test, ANOVA, Pearson coefficient of correlations, and through SPSS software with considering *P* < 0.05 as statistically significant.

## 3. Results

**Figure 1:**
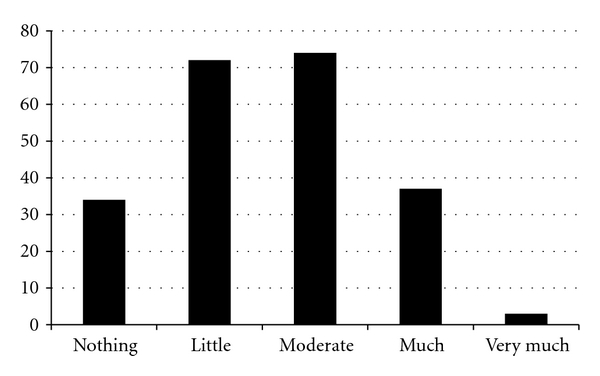
Impact of acne on quality of life patients according DLQI scores.

In the present work, 220 patients with acne were studied, of whom 181 (82.3%) were female. Mean ± SD age of patients was 22.05 ± 4.38 years and the youngest was 13 years old and the oldest was 33 years old. Mean disease duration was 4.15 ± 3.32 years and mean score of GAGS was 18.47 ± 7.28. Based on acne severity classification, mild acne had the highest frequency (51.8%) (Table 1). Mean ± SD DLQI score was 6.42 ± 4.77, and based on DLQI score classification, the impact of acne on quality of life was very much, much, and moderate in 114 patients (51.8%) and little or nothing in 106 (48.2%) patients (Figure 1). Mean score of CADI was 5.97 ± 2.97 ranging 0–14. Half of the subjects had CADI score of 6 or less.

In DLQI, the highest mean score was attained for disease symptom followed by feelings and emotions, and in CADI, body image has gained the highest mean score.

Statistical analysis showed significant difference in quality of life based on educational level, in a way that subjects with university degrees had better quality of life in comparison to other subjects (*P* < 0.01) whereas there was no significant difference in quality of life based on gender and marital status.

There was significant positive relationship between disease severity (GAGS) and scores of DLQI and CADI (*r* = 0.32, *P* < 0.01, and *r* = 0.28, *P* < 0.01), but no significant relationship was observed between the quality of life and the disease duration (Table 2).

## 4. Discussion

The results of the present study show clear impact of acne on patients' quality of life, and as it was mentioned acne had affected quality of life of 51.8% of subjects. The impact was very severe in 1.4%, severe in 16.8%, and moderate in 33.6% of the patients. CADI score was 5.97 in this study, while the results reported in similar studies in other countries are different. In Walker study in Scotland performed on students, mean score of DLQI and CADI has been, respectively, 1.7 and 1.9 [8]. In Hanisah study on Malaysian students, maximum CADI score has been 13 with medium of 4 [15]. Mean CADI score has been 2.56 in Law study in Hong Kong [16]. The quality of life of patients was been better in all three mentioned studies in comparison to the present study. One of the reasons for this difference may be related to our sampling process, which was based on those referring to dermatology clinics. It is clear that those referring to a doctor for acne may have more sever disease and/or different illness behaviour than others. On the other hand, in Abdel-hafez study in Egypt mean DLQI score has been 11.9 and 15.0 in female and male subjects, respectively, that are worse in comparison to the results of our study [17]. These differences may be due to the difference in disease severity, cultural differences, and also difference in individuals' expectations. 

There was not so strong relationship between quality of life (based on DLQI and CADI scores) and acne severity (based on GAGS). The results of other studies vary to some degree in this regard. While the relationship has been poor in Law study [16] and clear in Hanisha study [15], there has not been any relationship between the quality of life and disease severity in Yazici, Kokandi, and Ilgen studies [3, 18, 19]. It is definite that since quality of life is completely a subjective issue, the severity of lesions cannot determine the quality of life and some other factors are involved, such as social, personal, emotional, and school problems [19]. In the present study, there was no significant relationship between quality of life and duration of diseases. This result emphasizes the point that the effect of acne on quality of life is independent of disease duration and is mostly dependent on personal characteristics and patients' ability in accepting their disease and copying with its problems. The results of Kokandi study have been similar in this regard [3]. 

There was no significant difference in the quality of life based on gender that is similar to the results of Hanisha and Yazici studies [15, 18]. In Law study, quality of life has been better in males compared to females [16], while in Abdel-Hafez it has been the opposite [17]. 

Comparison of quality of life based on educational level showed better quality of life in those with higher educational levels that may be due to the social status of educated people enabling them to accept their disease and cope with its outcomes. 

It is noteworthy to mention that this study may be affected by selection bias because our sampling framework was limited to dermatology clinics rather than general population. However, Acne vulgaris had a significant impact on Iranian patients' quality of life. Clinician must consider the psychosocial impact of acne in addition to pharmacological treatment.

## Figures and Tables

**Table 1:** Sociodemographic and clinical characteristics of the patients (*n* = 220).

Variable	
Age (year), mean (SD)	22.05 (4.38)
Sex, No. (%)	
Male	39 (17.7)
Female	181 (82.3)
Marital status, No. (%)	
Single	158 (71.8)
Married	62 (28.2)
Level of education, No. (%)	
Diploma & Below	96 (43.6)
Academic	124 (56.4)
Disease Duration (year), mean (SD)	4.15 (±3.32)
Acne Severity (Based on GAGS)	
Mild	114 (51.8)
Moderate	93 (42.3)
Severe	13 (5.9)
Very severe	0

**Table 2:** Correlation between quality of life scores, duration, and severity of acne.

	DLQI	CADI	GAGS	Acne Duration
DLQI	1			
CADI	0.749*	1		
GAGS	0.315*	0.277*	1	
Acne Duration	−0.075	0.015	0.1	1

*Correlation is significant at the 0.01 level.
